# Customizable 3D-Printed (Co-)Cultivation Systems for In Vitro Study of Angiogenesis

**DOI:** 10.3390/ma13194290

**Published:** 2020-09-25

**Authors:** Ina G. Siller, Niklas-Maximilian Epping, Antonina Lavrentieva, Thomas Scheper, Janina Bahnemann

**Affiliations:** Institute of Technical Chemistry, Leibniz University Hannover, Callinstraße 5, 30167 Hannover, Germany; siller@iftc.uni-hannover.de (I.G.S.); epping@iftc.uni-hannover.de (N.-M.E.); lavrentieva@iftc.uni-hannover.de (A.L.); scheper@iftc.uni-hannover.de (T.S.)

**Keywords:** additive manufacturing, co-cultivation, mammalian cell culture, angiogenesis, biomaterials, biomedical application

## Abstract

Due to the ever-increasing resolution of 3D printing technology, additive manufacturing is now even used to produce complex devices for laboratory applications. Personalized experimental devices or entire cultivation systems of almost unlimited complexity can potentially be manufactured within hours from start to finish—an enormous potential for experimental parallelization in a highly controllable environment. This study presents customized 3D-printed co-cultivation systems, which qualify for angiogenesis studies. In these systems, endothelial and mesenchymal stem cells (AD-MSC) were indirectly co-cultivated—that is, both cell types were physically separated through a rigid, 3D-printed barrier in the middle, while still sharing the same cell culture medium that allows for the exchange of signalling molecules. Biochemical-based cytotoxicity assays initially confirmed that the 3D printing material does not exert any negative effects on cells. Since the material also enables phase contrast and fluorescence microscopy, the behaviour of cells could be observed over the entire cultivation via both. Microscopic observations and subsequent quantitative analysis revealed that endothelial cells form tubular-like structures as angiogenic feature when indirectly co-cultured alongside AD-MSCs in the 3D-printed co-cultivation system. In addition, further 3D-printed devices are also introduced that address different issues and aspire to help in varying experimental setups. Our results mark an important step forward for the integration of customized 3D-printed systems as self-contained test systems or equipment in biomedical applications.

## 1. Introduction

One of the major goals in the field of tissue engineering is the generation of artificial tissue grafts for replacement and/or reconstitution of damaged tissues and organs [1]. Maintaining adequate oxygen and nutrient supply within the tissue graft poses a particular challenge—and the use of diffusion as the main underlying transport principle of soluble nutrients is often insufficient in this context (especially in larger and dense tissues) [2]. To overcome this diffusion limit and to facilitate a fine distribution, a well-organized vascular vessel network must be generated [3]. The development of the human vascular system is mainly controlled by two different mechanisms: vasculogenesis and angiogenesis. Vasculogenesis describes the formation of new vessels from endothelial progenitor cells. In contrast, the reorganization and remodelling of an existing vascular network is mostly driven by angiogenic processes [3]. Angiogenesis is defined as the formation and growth of new blood vessels from existing vessels, as well as the restructuring of the vascular network [3]. As such, angiogenesis plays a fundamental role in numerous regeneration processes within the human body—and, consequently, a key act in tissue engineering [4]. The process of angiogenesis is determined by several cell types and the surrounding matrix (in particular, the basement membrane). Endothelial cells are the primary cell type involved [5]. After endothelial cells received signalling and activation, key steps in the subsequent generation of new vessel structures include the degradation of basement membrane, as well as the proliferation and cell migration of endothelial cells before they form and stabilize tubular structures and loops [5,6]. The alignment and arrangement of endothelial cells in tubes is referred to as tubular-like structures in this study.

There are numerous methods for assessing angiogenesis—all of which vary drastically in the particular part of the angiogenic cascade to which they refer, and in their clinical relevance [7]. Whereas in vivo approaches possess high potential to capture the complex processes of the human body, in vitro assays tend to be less laborious, achieving faster results and providing great quantification possibilities [6,7]. However, although in vivo assays are more time-consuming, expensive and limited in quantification, their high clinical relevance makes them indispensable in many respects [7]. However, before taking the steps towards conducting in vivo tests, in vitro assays can be beneficial in providing valuable initial information about test conditions. Since in vitro angiogenesis assays often focus on only one particular step in the angiogenic cascade, they exhibit high potential to consider the specific effects, interactions and role of tested drugs in the process of angiogenesis [6,8].

Traditionally, in vitro cell culture assays have been performed using only one single cell type—although the cells’ natural environment in vivo often comprises of various cell types [9]. Even if cells of different cell types are not in direct contact in vivo, they may at least be able to communicate with other cell types [9]. A common attempt to imitate an in vivo environment is represented in the simultaneous cultivation of several cell types within in vitro co-cultures. In principle, co-culture systems allow the cultivation of two or more cell types with a certain degree of contact/communication facilitated between them [10]. Especially for studying angiogenesis, co-culture approaches frequently prove beneficial, since angiogenic processes are stimulated by intercellular factors [3,11]. A well-known known and clinically relevant co-culture model for analysis of angiogenesis involves the co-cultivation of mesenchymal stem cells and endothelial cells [12,13]. Mesenchymal stem cells possess an angiogenic potential supporting the formation of tubular-like structures and characteristics in endothelial cells by releasing angiogenic factors [12,13,14]. Therefore, these cell types were selected as co-culture models in this study. In general, co-culture models cover direct cultivations of different cell types, which enables both direct cell-cell contact and interaction as well as indirect co-cultivation approaches, where different cell types are physically separated but sharing one cell culture medium that permits an intercellular communication and exchange of molecules [10,15,16]. Different co-culture models are commercially available—including co-culture inserts for well plates which facilitate the cultivation of two cell types that are physically separated from each other in one cell culture medium (for example, those distributed by ibidi GmbH, Gräfelfing, Germany or Thermo Fisher Scientific, Waltham, MA, USA) [17]. Petri dishes are also commercially available that have a surface separated in two, three, or even four compartments by a small barrier—a design which allows for a shared use of cell culture medium (for example, those distributed by Thermo Fisher Scientific, Waltham, MA USA). Numerous experimental co-culture setups have been developed and found their application in virtually all biological disciplines [10].

For studying vascularization and angiogenesis, in vitro approaches include indirect co-cultivation in commercial transwell inserts via use of membranes or in micropatterned systems [18,19,20,21]. Especially useful for analysing endothelial tube formation, one basic method involves seeding cells onto a layer of gel matrix, which provides those cells with nutrients and stimulates angiogenesis by releasing angiogenic factors [11,22]. Common matrix substances in such systems are collagen, fibrin, and Matrigel^®^, whereas Matrigel^®^ comprises of a solubilized basement membrane (from Engelbreth-Holm-Swarm mouse sarcoma) and evolved to be a standard substrate material in all kind of angiogenic tube formation assays in co- or mono-cultures [11,23]. Carter et al., have taken another approach and developed a porous and permeable membrane, which is similar in thickness to the vascular basement membrane and functions as a barrier or growth surface in co-cultivations [24]. Intensive research on cell-cell interactions, communication, and behaviour has also been conducted in direct co-cultivation studies in vitro—for example, through the use of endothelial and stromal cells [22,25,26]. More recent studies in the field of regenerative medicine have aimed to mimic the in vivo environment by (co-)culturing cells in 3D hydrogel or matrix structures, as well as on scaffold materials and beads [22,27,28,29,30]. These methods show a great potential for imitating the in vivo situation but are often regrettably limited in their capacity to ensure efficient oxygen and nutrient supply within the hydrogel or scaffold. In addition, analysis of cell behaviour and interaction in three dimensions still poses a considerable challenge [22]. Several approaches have been undertaken in microfluidics in recent years in an attempt to address this problem [11,18,31].

Importantly, however, all of these studies require appropriate experimental equipment, test beds, or—in the case of microfluidics and scaffolds-suitable microfluidic chambers and scaffolding elements. Indeed, the vast majority of reported microfluidic chambers have been fabricated by laborious, traditional manufacturing processes that require special training and are particularly time-consuming. Unless they wish to fabricate their own chambers, researchers currently have no choice but to revert to commercially available test equipment. Nowadays, additive manufacturing technology does enable the rapid production of customized labware and high definition experiment-specific equipment—but limitations remain. Even though many 3D printing materials are commercially available, not all of them are suitable for biomedical applications, due to the lack of biocompatibility or sufficient surface properties, for example [32,33].

Against this backdrop, this study introduces a customized 3D-printed co-cultivation system for use with indirect co-culture assays. Together with further developed 3D-printed (co-)cultivation platforms, this study illustrates the high potential of the 3D printing material in question—not only for the in vitro study of angiogenesis, but also for potential integration in biomedical applications more generally. To analyse the applicability of the customized co-cultivation system in angiogenesis studies, mesenchymal stem cells and endothelial cells were indirectly co-cultivated in the system. The release of angiogenic factors into the cell culture medium is expected to result in the formation of tubular-like structures by the endothelial cells. Endothelial tube formation was monitored via microscopic observation which was facilitated by the transparent appearance of the 3D printing material. Recently published work already indicated the great potential of the material in question with respect to proliferation and biocompatibility studies with mesenchymal stem cells [34]. Here, we further illustrate other excellent application possibilities for this versatile and highly promising material.

## 2. Materials and Methods

### 2.1. Design, 3D Printing and Post-Processing

As a first step in the manufacturing process, a 3D computer-aided design model was constructed. 3D computer-aided design (CAD) software SolidWorks 2018 (Dassault Systems, Waltham, MA, USA) was used for the design of all 3D-printed devices. The constructed CAD model was then directly sent to the 3D printer, where it is fabricated. A rigid, translucent, clear polyacrylate resin named AR-M2 (Keyence Deutschland GmbH, Neu-Isenburg, Germany) was selected as the 3D printing material, and processed using high-resolution 3D printer AGILISTA-3200 W (Keyence Deutschland GmbH, Neu-Isenburg, Germany). This printer manufactures objects via inkjet technology using an ultraviolet (UV) curing process, which results in a layer thickness of 15 µm and a resolution of 635 × 400 dots per inch.

Since surrounding supporting material must ultimately be removed from 3D-printed parts, a final “post-processing” phase is required after the printing phase is completed. Because the support material used (AR-S1; Keyence Deutschland GmbH, Neu-Isenburg, Germany) is soluble in water, the printed parts were placed for at least 1 h in a pre-warmed (60 °C) ultrasonic water bath (Bandelin electronic, Berlin, Germany) filled with deionized water and detergent (Fairy Ultra Plus, Procter and Gamble, Petit-Lancy, Switzerland), which was sufficient to remove all support material. After washing the parts thoroughly with deionized water, they were then “post-cured” with UV light (UV Sterilization Cabinet KT-09DC, Alexnld, Tiberias, Israel) for 1 h, in order to ensure full photo polymerization of the materials acylate monomers.

Immediately prior to its deployment in the biological environment, all 3D-printed parts were chemically disinfected via incubation in ethanol (70%, *v*/*v*) (Carl Roth GmbH und Co. KG, Karlsruhe, Germany) for 1h; then placed on a sterile surface for 1 h to allow the ethanol to evaporate; and, finally, washed thoroughly with sterile phosphate-buffered saline (PBS) (Carl Roth GmbH und Co. KG, Karlsruhe, Germany).

As highlighted in previous published work, the 3D printing material in question has a translucent clear appearance which facilitates optical microscopic observations. Known material components in the liquid state of the polyacrylate are two acrylate monomers, a photoinitiator, a stabilizer, and a urethane-acrylate-oligomer [34].

### 2.2. Cell Lines and Cell Culture Conditions

Two different cell lines were used in this study: GFP Human Umbilical Vein Endothelial Cells (HUVECs), and human adipose tissue-derived mesenchymal stem/stromal cells (AD-MSCs). HUVECs were purchased from Cellworks (Caltag Medsystems Limited, Buckingham, UK) and cryopreserved until usage. These cells are an angiogenesis co-culture that has been validated by the manufacturer. They were cultivated in basal Endothelial Cell Growth Medium 2 (EGM-2), with added supplement mix as instructed by the manufacturer (Promocell, Heidelberg, Germany), 8% fetale kalv serum (FKS) (Sigma Aldrich Chemie GmbH, Munich, Germany) and 0.5% gentamycin (PAA Laboratories GmbH, Pasching, Austria), at 37 °C in a 5% CO_2_ and 21% O_2_ humidified atmosphere. The AD-MSCs were isolated from adipose tissue following abdominoplasty surgery, extensively characterized as AD-MSCs, and cryopreserved in passage two until usage [35]. The donor provided informed written consent as approved by the Institutional Review Board (Hannover Medical School), with the reference number 3475–2017. For cultivation of AD-MSCs, a cell culture medium containing Minimum Essential Medium Eagle with alpha modification (α-MEM) (Thermo Fisher Scientific Inc., Waltham, MA, USA), 10% human serum (c.c.pro GmbH, Oberdorla, Germany) and 0.5% gentamycin (PAA Laboratories GmbH, Pasching, Austria) was prepared, and the cells were cultivated at 37 °C in a 5% CO_2_ and 21% O_2_ humidified atmosphere. Both cell lines were sub-cultivated with a confluence of 80–90% via accutase treatment (Merck KGaA, Darmstadt, Germany). All experiments were performed with cells of passages lower than nine.

### 2.3. CellTiter Blue (CTB) Viability Assay

The CellTiter Blue^®^ (CTB) cell viability assay was performed according to the manufacturer’s protocol (Promega GmbH, Mannheim, Germany)**.** In brief, cell culture medium was removed from each sample under examination, and 200 µL of fresh cell culture medium containing 10% CTB stock solution was then added, respectively. Samples were subsequently incubated at 37 °C in a 5% CO_2_ and 21% O_2_ humidified atmosphere for 1.5 h, and fluorescence signal was thereafter measured using a fluorescence plate reader (Fluoroskan Ascent, Therma Fisher Scientific Inc., Waltham, MA, USA). Viable, metabolically active cells are able to convert resazurin—which is contained in the reagent solution—into the fluorescent product resorufin. This conversion to resorufin can be monitored at an extinction wavelength of 544 nm and an emission wavelength of 590 nm.

### 2.4. Lactate Dehydrogenase (LDH) Based Viability Assay

Using the Cytotoxicity Detection Kit (Roche, Basel, Switzerland), the release of lactate dehydrogenase (LDH) into the culture supernatant was measured. Lactate dehydrogenase is a cytosolic enzyme which is released upon cell lysis or damage of the cell membrane. That way, the assay can be used to determine cell viability. The amount of LDH in the culture supernatant is measured by a coupled enzymatic reaction that causes tetrazolium salt to convert into a red formazan. The red formazan product holds an absorption maximum at 500 nm, which can be monitored. The assay kit was performed as specified by the manufacturer’s protocol, and a spectrophotometric microplate reader (BioTek Instruments, Inc., Winooski, VT, USA) was used to measure formazan formation.

### 2.5. Cell Proliferation Studies

Cell proliferation was monitored by performing the Trypan blue exclusion method. The Trypan blue stain is able to enter cells with compromised membrane integrity (i.e., damaged or lysed cells) and marks them with a blue colour. This allows for the differentiation between living and damaged/dead cells. After staining of the cells with 0.4% Trypan blue stain, the cell suspension is then pipetted in a haemocytometer (Brand GmbH + Co. KG, Wertheim, Germany), and a count of blue cells is taken.

Viability and proliferation studies of AD-MSCs and HUVECS growing on 3D printing material were performed as described in detail in previous published work [34]. While AD-MSCs were seeded in 3D-printed wells at a density of 15,000 cells·cm^−2^, HUVECs were seeded at a density of 25,000 cells·cm^−2^ (since these cells are smaller).

### 2.6. Co-Cultivation of AD-MSCs and HUVECs, Separated through Dividing Barrier

**Figure 1:**
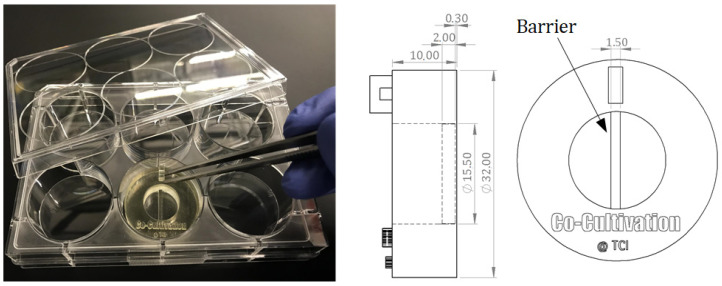
Illustration of a 3D-printed co-cultivation system that is separating two cell types through a dividing barrier. Both cell types can still share one cell culture medium and thereby enable exchange of substances. Picture of the 3D-printed system placed in a commercial well plate (**left**); CAD drawings of the co-cultivation system with dimensions. All dimensions in CAD drawings are in mm (**right**).

For indirect co-cultivation experiments, a cell cultivation system was designed that allows for separate cultivation of two adherent growing cell types within a single shared cell culture medium. The growth surface area is divided into two spaces by a rigid barrier wall. Each side represents the surface area for cell adhesion and growth of one cell type. The 3D-printed barrier provides a sufficient height (2 mm) to prevent cell-cell contact among the different cell types but still simultaneously enable both sides to share the same cell culture medium. This allows endothelial cells (here: HUVECs) to be physically separated from stromal cells (here: AD-MSCs), and to develop tubular-like structures due to stimulating substances released from feeder cells. The separate cultivation of AD-MSCs and HUVECs using a physical barrier within a single shared cell culture medium is referred to as “indirect co-cultivation” in this study. The designed co-cultivation system fits in a well of a commercially available 6-well cell cultivation plate, which provides sterile and user-friendly handling. Cultivation surfaces for adherent cell growth of two cell types are separated through a barrier. The CAD-design, dimensions, and the handling are all illustrated in Figure 1.

Following 3D printing, post-processing, disinfection, and an evaporation procedure, the co-cultivation systems were washed thoroughly with PBS. Both cell types were then seeded separately and independently from each other within a cell-type-specific culture medium. HUVECs were seeded at a density of 30,000 cells per side within the co-cultivation chambers, while AD-MSCs were seeded at a density of 20,000 cells per side. Cells were seeded in a volume of 200 µL cell culture medium. This volume allowed for discrete seeding, without overflowing the barrier. For cell seeding and adhesion, cells were kept in their regular cell-type-specific culture medium. After 24 h, the medium of both cell types was removed, and a co-cultivation medium was added. The co-cultivation medium contains half EGM-2 and half α-MEM, at 3% human serum and 0.5% gentamycin. A volume of 550 µL of co-cultivation medium permits an overflow and exchange of the medium across the physical barrier. The cultivations were maintained at 37 °C in a 5% CO_2_ and 21% O_2_ humidified atmosphere. Cell growth, behaviour, and angiogenesis were all subsequently monitored using a cell imaging multi-mode reader (Cytation 5; BioTek Instruments, Inc., Winooski, VT, USA).

### 2.7. Co-Cultivation of AD-MSCs and HUVECs without Physical Separation

For further co-cultivation and screening approaches, co-cultivations of AD-MSCs and HUVECs in which both cell types were not physically separated from each other were also performed. The collective cultivation of AD-MSCs and HUVECs without a physical barrier is referred to as “direct co-cultivation” in this study. Within this experimental setup, AD-MSCs and HUVECs were seeded in different cell mixture ratios in a screening assay. A screening platform was designed and 3D-printed, and then fitting into a regular 60 mm petri dish, in order to facilitate sterile and user-friendly handling. In a first approach, a cell count of 70,000 cells per cultivation well was set, and the correspondence ratios were respectively calculated. Chosen cell mixture ratios were 1:1, 1:2, 1:3, 1:5, 2:1, 3:1, and 5:1 (HUVEC:AD-MSC). The 3D-printed cultivation platform was maintained at 37 °C in a 5% CO_2_ and 21% O_2_ humidified atmosphere. Cell growth, behaviour, and angiogenesis were all subsequently monitored using a cell imaging multi-mode reader (Cytation 5; BioTek Instruments, Inc., Winooski, VT, USA).

### 2.8. Crystal Violet Staining Method

All attached cells were marked using a method of crystal violent staining. Crystal violet is a triarylmethane dye that can bind to DNA and proteins [36]. For staining, the cell culture medium was removed, and the cells themselves were washed with PBS. 200 µL of a prewarmed crystal violet staining solution (Merck KGaA, Darmstadt, Germany), which was added to each well and incubated for 10 min at room temperature. Afterwards, the staining solution was removed, and the chambers thoroughly washed with PBS. Microscopic images were then taken with a 3D digital microscope (VHX; Keyence Deutschland GmbH, Neu-Isenburg, Germany).

### 2.9. Evaluation of Angiogenesis

To detect and quantitative analyse angiogenic tube formation, the free open source software AngioTool^®^ (National Institute of Health, National Cancer Institute, Bethesda, MD, USA) was utilized [37]. For analysis of experiments, AngioTool^®^ can detect the output of various morphometric parameters related to angiogenesis (such as the vessel area, total number of junctions, or the average vessel length). Vessel profiles and networks were then identified according to the parameters set. In this study, fluorescence images were analysed using the following settings present in AngioTool^®^: vessel diameter = 12 µm; vessel intensity = 15–255; and fill holes = 240. These parameters were selected because they had previously proven to be suitable.

For better imagination, a figure demonstrating the output of AngioTool^®^ and a detailed description of analysis parameters are given in the Supplementary Materials (see Figure S1, Supplementary Materials).

## 3. Results and Discussion

### 3.1. Viability and Growth Analyses of Cells Growing on 3D Printing Material

Guaranteed biocompatibility is an essential prerequisite for all materials that are introduced to a biological environment (for example, within biomedical applications). Any potential negative effects on cells—for example, via leaching of substitutes or degradation of products—must be definitively ruled out, since these characteristics can induce irritations and/or allergic reactions [38,39,40].

Recently published work in studies using AD-MSCs had already demonstrated the general biocompatibility of the 3D printing material in question [34]. As co-cultivations are generally performed with various cell types, further biocompatibility studies of the printing material with HUVECs were also conducted. Therefore, AD-MSCs—just as HUVECs—were cultured in direct contact with the 3D printing material, and cell viability, morphology, and proliferation were all carefully monitored. HUVEC cell viability and proliferation was compared to AD-MSCs viability, as well as to the cell growth on the cell culture-treated surfaces of commercially 24-well plates (as a control). For these experiments, cell cultivation chambers were 3D printed. The cultivation chambers are designed to fit into the well of a regular 6-well cell cultivation plate, which helped to ensure sterile and user-friendly handling. By adapting the growth surface area of the 3D-printed cultivation chamber to the growth surface area of a commercial 24-well cell cultivation well, control cultivations can be performed using commercially available 24-well cell cultivation plates. The growth surface area of both of the cultivation systems that were used was 1.89 cm^2^. Here, cultivations in commercial 24-well cell cultivation plates represent the optimal cell growth with optimal conditions.

**Figure 2:**
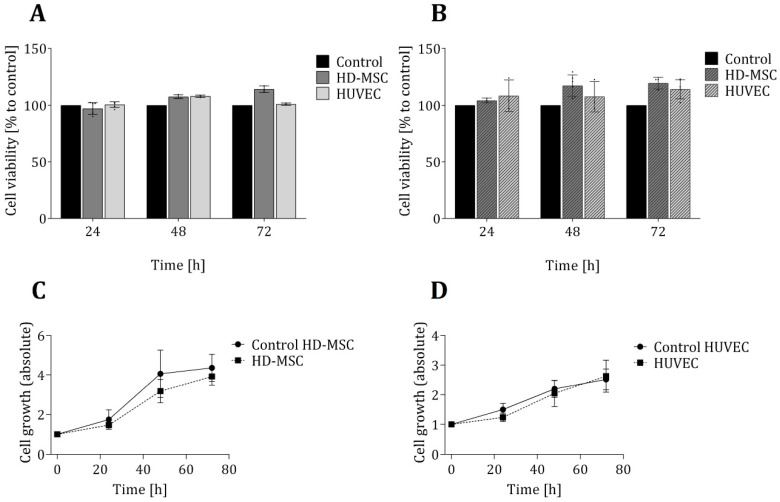
Evaluation of cell viability and proliferation of cells growing in direct contact to the 3D printing material. Cell viability of both cell types (AD-MSCs and HUVECs) was analysed in LDH assays (**A**) and CTB assays (**B**) after cultivation of cells for 24 h, 48 h and 72 h in 3D-printed chambers. Control cultures of both cell types were performed in commercially available regular 24-well cell culture plates and are considered to present cell growth under optimal conditions. Cell viability is normalized to the corresponding control, respectively. Cell growth was determined by cell counting of living cells using Trypan blue staining and illustrated for HD-MSCs (**C**) and HUVECs (**D**). All experiments were repeated several times (*n* > 6) and compared to control.

In order to ensure that the 3D-printed material in question has no toxic effects on the cells, two biochemical-based in vitro viability assays were also performed—each based on a different metabolic activity and function in the cellular metabolism (see Section 2.3 and Section 2.4). Figure 2 shows the results of these viability assays, as well as proliferation studies of both cell types that were grown directly on the 3D-printed material. No significant differences in cell viability for either cell types (in comparison to the control cultures) were observed. In fact, over a cultivation period of 72 h, the average cell viability observed within the 3D-printed chambers was slightly higher than that observed within the commercial 24-well plates. Furthermore, an analysis of cell growth showed no significant differences in growth rates for either cell types—regardless of whether cells were cultivated in 3D-printed systems or in commercial cell cultivation well plates.

The transparent clear appearance of the 3D-printed material also allows for optical microscopic monitoring of the cell morphology. Such observation did not reveal any changes in either the cell morphology or behaviour for cells that were cultivated in direct contact with the 3D-printed material. Taken together, neither cell viability and proliferation nor morphology appears to have been influenced by the 3D printing material for either of the cell types that were analysed.

### 3.2. Co-Cultivation of HUVECs and AD-MSCs in 3D-Printed Cell Cultivation Systems

A common approach to mimicking an in vivo environment for the purposes of studying intercellular interactions is the in vitro cultivation of different cell types in co-cultures, which implies a simultaneous cultivation of several cell types. There are many such approaches: For example, direct co-cultivation systems enable both cell-cell contact and interaction between different cell types. By contrast, in indirect co-cultivations, the different cell types are physically separated but still share one culture medium, which allows for the exchange of signalling molecules via the medium.

3D printing represents an ideal, flexible tool to satisfy the widely varying demands of different experimental setups. For an indirect co-cultivation of AD-MSCs and HUVECs, a cultivation system was designed and 3D-printed (see Figure 1). The cavity in the middle was divided into two spaces by a rigid, 3D-printed barrier. Each side represents the surface area for cell adhesion to facilitate the growth of one cell type. The barrier was high enough to physically separate the two cell types but still simultaneously allow both sides to share a single cell culture medium. To maintain a sterile environment while still enabling user-friendly and convenient handling, the dimensions of the co-cultivation system were also adapted to fit in the well of a commercially available 6-well cell cultivation plate (see Figure 1).

**Figure 3:**
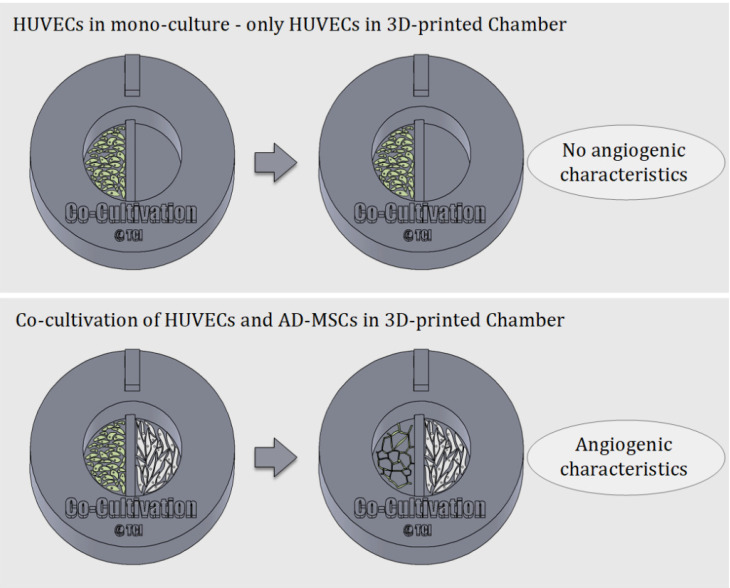
Schematic illustration of the underlying principle of indirect co-cultivation pointed out. While HUVECs cultured alone in the 3D-printed chamber show no signs of angiogenesis, HUVECs cultured in co-cultivation with AD-MSCs in 3D-printed chambers develop characteristics of angiogenesis.

One of the most well-known and clinically relevant in vitro co-culture models facilitates cultivation of mesenchymal stem cells and endothelial cells [12,13]. These models are frequently used (for example) to study the angiogenic potential of MSCs from different sources and donors, as one of the required potency assays [14,41,42]. Such assays evaluate MSCs supporting the formation of tubular-like structures of endothelial cells through release of angiogenic factors [13,42]. In this work, we analysed the suitability of using a 3D-printed co-cultivation platform for the development and assessment of endothelial tubes in the presence of AD-MSCs. Figure 3 demonstrates the principle of an indirect co-cultivation within the 3D-printed chamber. While HUVECs cultured in mono-culture do not display characteristics of angiogenesis, HUVECs cultured in a shared medium alongside AD-MSCs form tubular-like structures that are considered a characteristic of angiogenesis.

**Figure 4:**
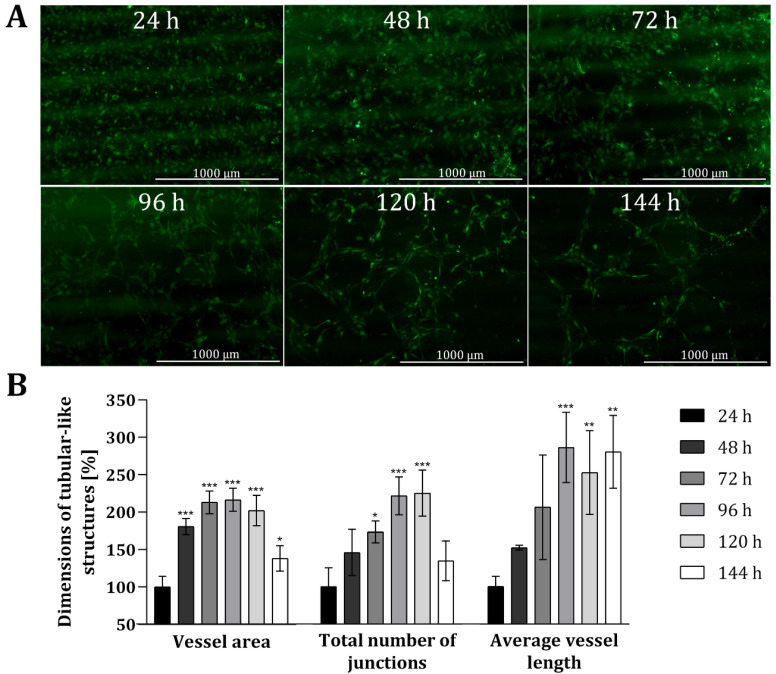
Analysis of angiogenic tube formation in indirect co-cultivation in 3D-printed system. (**A**): Fluorescence images of HUVECs cultivated in co-cultivation with AD-MSCs, physically separated through a barrier. After 24 h, the medium was changed to co-cultivation medium. In all, cells were co-cultivated for 144 h. (**B**): Results of quantitative analysis of the vessel area, total number of junctions and average vessel length of tubular-like structures of HUVECs using the software AngioTool^®^. HUVECs cultured in monoculture in 3D-printed system served as negative control. All experiments were repeated several times in independent experiments (*n* > 3) and significant differences to negative control are indicated: * *p* < 0.05. ** *p* < 0.01, *** *p* < 0.001.

To analyse the suitability of the 3D printing material, as well as the customized 3D-printed co-cultivation system in the context of indirect co-cultivation approaches, co-cultures of HUVECs and AD-MSCs were conducted over a period of 144 h (6 days, and endothelial tube formation was carefully monitored. In this study, all experiments were performed with HUVECs expressing green fluorescent protein (GFP) to facilitate monitoring via fluorescence. Because the 3D printing material in question holds no notable degree of autofluorescence, it readily permitted microscopic monitoring of fluorescence. Figure 4 shows fluorescence images of HUVECs grown in the 3D-printed co-cultivation chamber taken over a cultivation period of 144 h, as well as a quantitative analysis of tubular-like structures. Both cell types were seeded independently in their cell specific cell culture medium, in a volume that allowed for discrete seeding without overflowing the barrier in the middle. After a cultivation period of 24 h, culture media were removed and changed to co-cultivation medium in a volume that permitted the indirect interaction of both cell types via the transmission of signalling molecules.

The obtained fluorescence images illustrate that a tubular network was, indeed, formed in the course of time (see Figure 4A). Already after 48 h the slow migration and the associated accumulation of HUVECs can be seen, as smaller gaps are formed in the cell layer. These gaps become much wider after 72 h and over time, as the HUVECs accumulate more and more in a tubular structure. The cells gradually develop into a tubular-like network with increasing vessel or tube length, while the thickness of the formed tubes is declining. Quantitative analyses also revealed that the dimensions of tubular-like structures increased over time (see Figure 4B) when compared to HUVECs only cultured in mono-culture within 3D-printed systems. The dimensions of the vessel area, the total number of junctions, and the average vessel length was more than doubled after around 72 h. The average length of the vessel in particular notably continued to extend as time progressed (i.e., to 286% ± 47% after 96 h, and ultimately to 280% ± 48% after 144 h). By contrast, the vessel area and total number of junctions both decreased again after 144 h—with a *p*-value of 0.0399, the dimensions of the vessel area are only slightly significant different, and no significant difference was observed for the total number of junctions compared to the dimensions after 24 h. Fluorescence images taken after 144 h of co-cultivation also highlighted that the tubular-like networks formed wide holes, while the thickness of the developed tubes declined. One potential reason for the reduced vessel area and thickness of tubular structures after 144 h could be due to the loss of cells via detachment of dead cells.

In all, this experiment demonstrates that endothelial cells form tubular-like networks within the context of an indirect co-cultivation approach with AD-MSCs in a customized 3D-printed system. There was no need of applying Matrigel^®^ or any other substrate materials to stimulate angiogenesis; rather, due to the angiogenic potential of AD-MSCs, and a potential release of angiogenic factors in the cell culture medium, co-culture alongside AD-MSCs facilitated the development of angiogenic features within endothelial cells [4]. The process of angiogenic tube formation may of course be accelerated by applying matrix substances (which could easily be coated onto the 3D-printing material). The material used for 3D printing has also been proven to be suitable for cell cultivation—particularly in the context of this customized 3D-printed co-cultivation system. This could represent a promising starting point for further investigations of intercellular interactions, or for assessing the effects of potential regulators on vessel formation. To the best knowledge of the authors, this experiment marks one of the first indirect co-cultivation assay in this vein that has been performed within a 3D-printed system—and thereby represents an important step towards the integration of customized 3D-printed devices in biomedical and/or tissue engineering applications [43,44].

### 3.3. Further Customizable, Experiment-Specific 3D-Printed Platforms for the Study of Angiogenesis

In vitro angiogenesis studies provide valuable initial information about (for example) drug substances, suitable test concentrations, and cell therapy candidates, to name just a few applications. Essential test variants can be performed in vitro before considering their use in in vivo experiments. In addition, in vitro angiogenesis assays often address only one particular step in the angiogenic cascade—such as the degradation of basement membrane or endothelial cell proliferation and migration—and thus hold the potential to provide relevant information about specific interactions, or classify roles of tested drugs in a particular phase of the angiogenesis process [6]. For these reasons, there are numerous studies reporting on in vitro angiogenesis assays [7,22].

**Figure 5:**
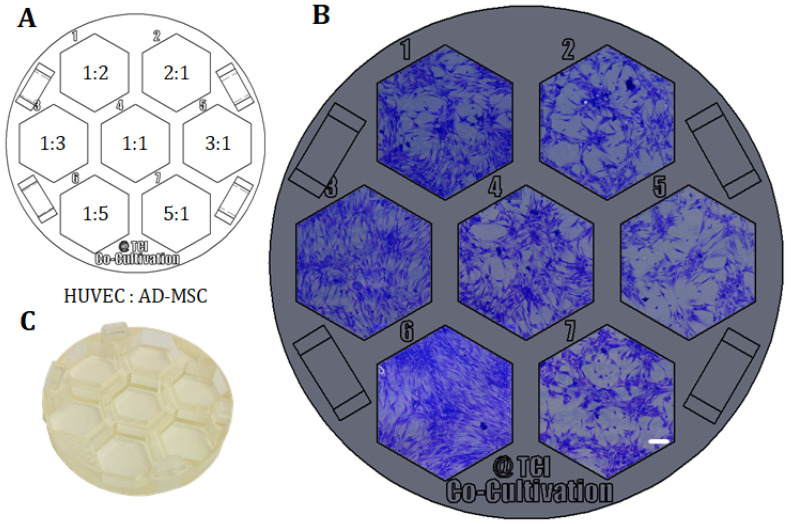
Example of direct co-cultivations (without physical barrier) performed in a 3D-printed screening platform fitting in a commercial petri dish. HUVECs and AD-MSCs were seeded in different cell mixture ratios (1:2, 1:3, 1:5, 1:1, 2:1, 3:1, 5:1), while cell density per well was held constant and co-cultivated in co-cultivation medium for 96 h. (**A**): Different cell mixture ratios of HUVECs and AD-MSCs as seeded in the screening platform. (**B**): Images of co-cultivations with cells seeded in different cell mixture ratios in 3D-printed system. Images were taken with a 3D digital microscope after crystal violet staining; scale bar is 150 µm. (**C**): Picture of the 3D-printed platform, printed with the 3D-printing material named AR-M2 (Keyence Deutschland GmbH, Neu-Isenburg, Germany).

**Figure 6:**
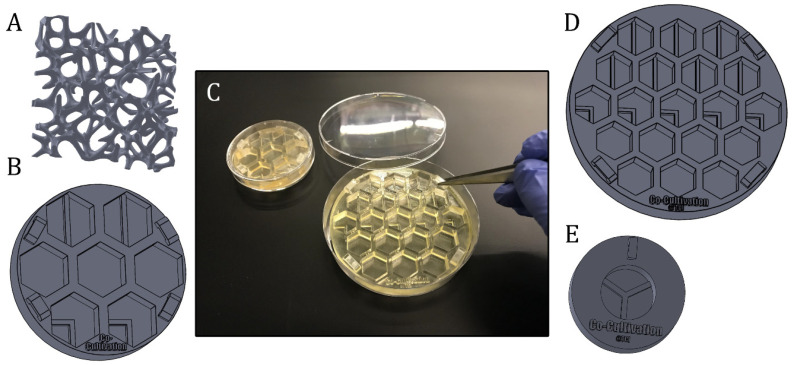
Presentation of different experiment-specific co-cultivation platforms and approaches. (**A**): Scaffold structure that can directly be seeded with cells, coated or filled with matrix substrates (**B**): Screening platform, which is fitting in a regular petri dish Ø 60 mm. (**C**): Illustration of the handling of 3D-printed screening platforms, which are fitting in commercial petri dishes with diameters of 60 mm or 90 mm. (**D**): Example of a co-cultivation platform with 19 wells for screening approaches. The system is fitting in a regular petri dish with a diameter of 90 mm. (**E**): Co-cultivation system allowing separate growth of three different cell types, while sharing still one cell culture medium.

However, general in vitro test setups and requirements in research can vary widely between experiments. Suitable experimental equipment, test beds, or supporting scaffold structures may be needed—which can pose a significant challenge for researchers. Unless they are prepared to produce their own labware, researchers are dependent on commercially available products. At this point, 3D printing technology represents an advantageous tool to fabricate individually and experiment-specific products in high-resolution and with almost unlimited complexity. Some possible applications of 3D-printed systems supporting in vitro studies (of angiogenesis) are illustrated in Figure 5 and Figure 6.

One example of how 3D printing can be used to create new opportunities or circumvent limitations imposed by existing commercially-available equipment is demonstrated in the following. As a proof of concept, a platform with seven cell cultivation wells was specifically designed and 3D-printed for a direct co-cultivation approach with endothelial cell (HUVECs) and stromal cells (AD-MSCs). For the direct co-cultivation, the wells were not divided by a barrier and, consequently, the two cell types were cultivated with direct cell-cell contact. This custom 3D-printed system was then fitted into a commercial petri dish (Ø 60 mm) as pictured in Figure 6C, to ensure sterility and user-friendly handling of the 3D-printed platform. It must be emphasized that the chosen design format represents only one of many design possibilities and shows the great potential of the 3D printing technology used in this study for cell culture applications.

With the goal of finding the most promising cell mixture ratio, HUVECs and AD-MSCs were thereafter seeded into the cell cultivation wells in different ratios, while the cell density per well was held constant. Here, variation of cell mixture ratios led to different degrees of formation of holes and tubular-like structures as angiogenic features—probably in response to different local concentrations of pro- or anti-angiogenic signalling molecules secreted by AD-MSCs. In this experimental approach, a high ratio of AD-MSCs caused overgrowth or domination over the endothelial cells as the whole cell cultivation well became pervaded by AD-MSC-typical cell layer morphologies (see cell mixture ratios of 1:5 and 1:3 in Figure 5). In conclusion, a cell mixture ratio of 2:1 (HUVEC: AD-MSC) was determined to be advisable for future experiments.

This experiment illustrates what a useful tool 3D printing technology can be for meeting experiment-specific challenges. As long as the 3D printing material in question is biocompatible and can also fulfil all experimental requirements with respect to mechanical and optical material properties, it can function as a self-contained test system; supporting scaffold structures for 3D cell culture experiments; individually-designed labware; or even a high-throughput system and cell culture vessel.

Figure 6 illustrates further variations of 3D-printed (co-) cultivation platforms and approaches designed to study angiogenesis in vitro. These screening platforms were again fitted in commercial petri dishes to function as a test bed—with the possibility to apply Matrigel^®^ or other hydrogel matrix substances as a supporting base, if desired—and proved helpful in finding a suitable test concentration of a pro- or anti-angiogenic drug in more complex 3D culture systems. Screening platforms with co-cultivation wells—i.e., where the growth area is divided through a rigid barrier—may help to facilitate the study of angiogenic potential of stromal cells from different sources and donors (see Section 2.2). 3D-printed Co-culture systems with printed barriers dividing the growth area into thirds enable the separate growth of three different cell types even as the cells continue to still share one cell culture medium.

Another attribute that is known to affect angiogenesis is the topography of the material’s surface with which the cells are in contact [45,46]. Scarano et al., were able to show that the formation of blood vessels is stimulated by concavities integrated in the material structures [45]. Furthermore, Yang et al., demonstrated the influence of micro/nano structured surfaces on osteogenesis and angiogenesis [46]. The different hierarchical structures regulated the inflammatory response of macrophages, which also affected osteogenesis and angiogenesis [46]. Thus, by adjusting the design of the material or, for example, the implant, the formation of blood vessels and ultimately the implant integration and acceptance could therefore be optimized. Similar approaches could potentially be transferred to 3D-printing applications, since the introduction of high-resolution 3D-printing enables the fabrication of detailed and complex structures—even in micro and nanoscale format.

The provision of adequate nutrient and oxygen supply within a tissue graft continues to constitute a significant challenge in the field of tissue engineering [3]. Indeed, overcoming limitations on diffusion is a major goal in modern graft engineering [3]. In order to achieve this, both the physiologic and pathologic appearance of the vascular system must be carefully analysed and replicated [47]. In an attempt to mimic vascular networks, Kang et al., fabricated porous scaffolds via stereolithography, coated them with a collagen layer, seeded cells onto the matrix, and then integrated the construct in a perfusion system to imitate blood flow through the scaffold [47]. Similar approaches could potentially be pursued and enhanced via 3D-printed scaffolds printed with the material used in this study. Scaffold structures of almost unlimited complexity can be 3D-printed, and cells can be grown directly on the scaffold, on coating matrix substances (e.g., hydrogels or Matrigel^®^), or in hydrogel substances in pores. The whole system can then be integrated into a flow chamber. Customizable 3D-printed platforms therefore hold tremendous promise to broaden the spectrum of in vitro systems available to researchers.

## 4. Conclusions

This study introduces a customized 3D-printed co-cultivation system useful for indirect co-cultivation approaches, as well as further 3D-printed devices useful for the in vitro study of angiogenesis. The results first confirmed that the 3D printing material does not exert any negative influences on cell behaviour. The customized 3D-printed co-cultivation system was then proven to be a suitable platform for performing and monitoring indirect co-cultivations. Angiogenic tube formation of endothelial cells was observed to develop within this system in response to the potential release of angiogenic factors by co-cultured mesenchymal stem cells—without the need for any supporting substrates (such as, for example, Matrigel^®^). Since the 3D printing material also enables phase contrast and fluorescence microscopy, the behaviour of cells could be observed over the entire cultivation period via both. To the best of the authors’ knowledge, there have been only few works published that demonstrates how 3D-printed systems can be used as customizable platforms in indirect 2D co-cultivation approaches [43,44]. We believe this paper therefore marks an important step forward for the integration of customized 3D-printed systems as self-contained test systems or equipment in biomedical applications.

Angiogenesis studies, and indeed cell culture work in general, frequently confront researchers not only with difficult scientific questions but also profound implementation challenges. Researchers are forced to find suitable assays and methods to address and assess complicated experimental problems. However, all too frequently researchers are already restricted even in the very first phase of problem-solving (i.e., formulating experimental design) because they are reliant on commercially available experimental equipment. The rise of experiment-specific, customized 3D-printed labware can remove this artificial and regrettable limitation. Moreover, high-resolution 3D printing technology can open the door to manufacturing even complex devices at a micro scale [48]. Personalized experimental equipment or whole cultivation systems of almost limitless complexity can potentially be produced from start to finish within just a few hours—offering for a tremendous potential for pursuing experimental parallelization within a highly controllable environment. The 3D printing material used in this study outshines and also convinces by its suitability in cell cultivation experiments, as it can be exposed to ethanol (without negative effect on the material or later on the cells). Furthermore, autoclavable 3D printing materials are also commercially available. Thus, the sterility of the 3D-printed parts can be assured. Moreover, the mechanical and optical properties of the 3D printing material used here make it a very promising candidate for producing customized cell culture products. Whether as customized test system (as highlighted in this study) or as a high-throughput screening platform, as scaffold material or as experiment-specific labware—the 3D printing material in question may have almost unlimited application in future research setups.

## Supplementary Materials

The following are available online at https://www.mdpi.com/1996-1944/13/19/4290/s1, Figure S1: Exemplary images of cell analysis using AngioTool^®^. Figure S1A: Fluorescent image of GFP Human Umbilical Vein Endothelial Cells (HUVECs) cultivated for 120 h in the 3D-printed co-cultivation system. Total magnification is 4. Figure S1B: Output of AngioTool^®^. All vessels that are considered by the software are marked in red and all branching points are highlighted in blue.
