# Supplementary material for: Customizable 3D-Printed (Co-)Cultivation Systems for In Vitro Study of Angiogenesis
Source: Materials (Basel). 2020 Sep 25;13(19):4290. doi: 10.3390/ma13194290 (PMC7579111; doi:10.3390/ma13194290)
Supplement: Supplementary file 1 [file materials-13-04290-s001.pdf]

materials

Supplementary Materials: Customizable 3D-Printed (Co-)Cultivation Systems for In Vitro Study of Angiogenesis

Ina G. Siller, Niklas-Maximilian Epping, Antonina Lavrentieva, Thomas Scheper and Janina Bahnemann *

Institute of Technical Chemistry, Leibniz University Hannover, Callinstraße 5, 30167 Hannover, Germany; siller@iftc.uni-hannover.de (I.G.S.); epping@iftc.uni-hannover.de (N.-M.E.); lavrentieva@iftc.uni-hannover.de (A.L.); scheper@iftc.uni-hannover.de (T.S.)
* Correspondence: jbahnemann@iftc.uni-hannover.de

Evaluation of Angiogenesis by using AngioTool® software

For quantitative analysis of angiogenesis, the open source software AngioTool® (National Institute of Health, National Cancer Institute, Bethesda, MD, USA) was used. Images were analysed regarding different parameters that are related to angiogenesis. As one of the parameters, the vessel area was analysed, which is defined as the area of all segmented vessels while a vessel is defined in AngioTool® as a segment between two branching points. Furthermore, the total number of junctions or branching points between vessels was determined as well as the average vessel length of all vessels in the image. Figure S1 illustrates the output of AngioTool® for an exemplary image. Additionally, an Excel sheet with the calculated results for the analysis parameters was issued by the software.

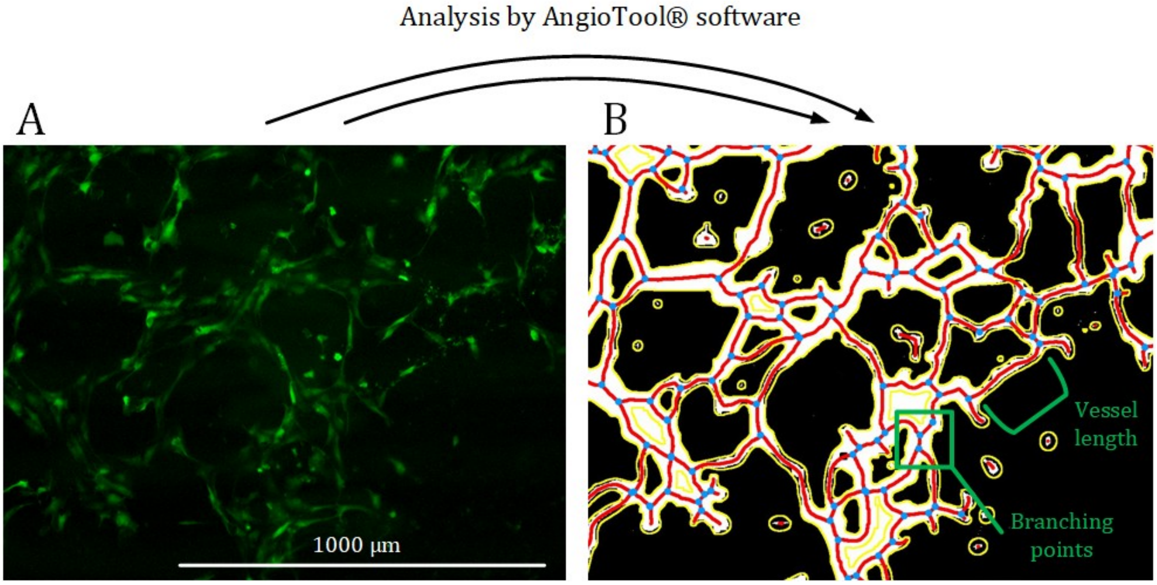

Figure S1. Exemplary images of cell analysis using AngioTool®. (A): Fluorescent image of GFP Human Umbilical Vein Endothelial Cells (HUVECs) cultivated for 120 h in the 3D-printed co-cultivation system. Total magnification is 4. (B): Output of AngioTool®. All vessels that are considered by the software are marked in red and all branching points are highlighted in blue.

© 2020 by the authors. Submitted for possible open access publication under the terms and conditions of the Creative Commons Attribution (CC BY) license (http://creativecommons.org/licenses/by/4.0/).
